# Chromosome-level genome and population genomics reveal demographic history, incomplete divergence and coastal adaptation of *Rhododendron simsii* var. *putuoense* in East China

**DOI:** 10.1016/j.pld.2025.12.002

**Published:** 2025-12-10

**Authors:** Hong Zhu, Haojie Gao, Hepeng Li

**Affiliations:** a Zhejiang Academy of Forestry, Research Centre for Zhejiang Wetland, Hangzhou 310023, China; b Zhoushan Academy of Forestry, Zhoushan 316000, China

**Keywords:** *Rhododendron simsii* var. *putuoense*, *de novo* assembly, Comparative genomics, Population genomics, Demographic history, Coastal adaptation

## Abstract

• First chromosome-level genome assembly of *Rhododendron* *simsii* var. *putuoense* (479.30 Mb, contig N50 = 33.97 Mb, 98.4 % BUSCO), advancing Ericaceae genomics.
• 21 populations form coastal-island genetic groups with incomplete divergence, moderate diversity, gene flow; island groups show stronger selection and inbreeding.
• Adaptive evolution via WGD, expanded secondary metabolite gene families, and positively selected salt/cold tolerance genes, revealing coastal multi-stress strategies.

Geographical isolation plays a crucial role in species divergence and adaptive evolution (Worsham et al., 2017; Loretán et al., 2020). This phenomenon is especially pronounced in land-sea interactive habitats, where geological shifts and climatic variations have significantly influenced genetic structures and distribution patterns by repeatedly modifying habitat connectivity (Jiang et al., 2021). Land-bridge islands, which extend from continental shelves and experience alternating phases of connection and isolation due to glacially driven sea-level fluctuations, function as natural laboratories for investigating species divergence, gene flow, and ecological adaptation (Jin et al., 2016; Cros et al., 2020). The Zhoushan Archipelago, China’s largest archipelago, located on the northern margin of the subtropical zone, has undergone similar geological dynamics (repeated geological linkages and separations from the mainland), forming a distinct “island-continent disjunct distribution” pattern. This characteristic makes it an ideal system to study plant responses to geographical isolation in East Asia (Zhang et al., 2025).

*Rhododendron simsii* var. *putuoense* G.Y. Li & Z.H. Chen 2010 (Fig. S1) is an endemic species of *Rhododendron* found in eastern China, restricted to the coastal regions of Zhoushan Archipelago and adjacent regions of Zhejiang Province (Li et al., 2010). It primarily inhabits coastal scrublands and rocky coastal forests below 400 m. This variety differs from the nominal form *R. simsii* by its earlier flowering period (occurring from early March to early April) and its rose-red corollas, whereas *R. simsii* typically exhibits dark red corollas and flowers from mid-March to May across a broader subtropical distribution in China. This coastal island distribution gives it horticultural potential and scientific value as a model for adaptive evolution in extreme coastal environments. The species’ dual distribution across island and adjacent mainland (coastal) populations offers a rare opportunity to explore how geographical isolation intensity influences genetic divergence.

Advances in high-throughput sequencing have facilitated genomic studies of species evolution (Ao et al., 2022; Jing et al., 2025). However, existing research on *Rhododendron* has focused predominantly on high-elevation taxa in western China (Xia et al., 2024), leaving genomic resources and population genetic mechanisms of East China coastal groups understudied. Although the taxonomic status of *R. simsii* var. *putuoense* has been preliminary confirmed (Zhu et al., 2025), critical gaps remain in understanding its genetic relationships with closely related species, divergence time, population genomic structure, and adaptive selection mechanisms. This lack of knowledge hinders a comprehensive understanding of species and the development of effective conservation strategies.

Notably, this dual island-coastal distribution provides a unique framework to analyze the interplay between geographical isolation, gene flow, and adaptive divergence. Yet its evolutionary history and mechanisms of local adaptation remain unclear. To address this, we generated the first chromosome-level genome of *Rhododendron simsii* var. *putuoense* via *de novo* sequencing and conducted resequencing analysis of 21 natural populations. These sequence data allowed us to characterize genomic structural characteristics and resolve phylogenetic relationships and divergence time with closely related species; disentangle genetic differentiation patterns and gene flow dynamics between island and coastal populations to assess the impact of geographical isolation on genetic structure; and identify adaptive selection mechanisms underlying coastal stress tolerance. This study fills critical gaps in coastal *Rhododendron* genomics, offers new insights into the early-stage divergence of land-bridge island plants, and provides a scientific basis for conserving this endemic species and sustainably utilizing its horticultural potential.

We generated a chromosome-level genome of *Rhododendron simsii* var. *putuoense* by integrating short-reads from the Illumina NovaSeq 6000 platform for chromosome conformation capture (Hi-C) technologies with PacBio Sequel II HiFi sequencing data. Long-read RNA sequencing was performed using the Nanopore PromethION 48 platform to support and validate the assembly results (Table S1). The assembly exhibited high quality: genome size 479.30 Mb, with contig N50 and scaffold N50 lengths of 33.97 Mb and 36.50 Mb, respectively. In total, 478.85 Mb (99.91%) of assembled sequences were anchored to the 13 pseudo-chromosomes, with lengths ranging from 32.06 to 44.64 Mb (Fig. 1a and Table S2). The heat map of Hi-C interactions shows that the genome assembly is intact and robust (Fig. S2), supported by a high LTR Assembly Index (LAI = 16.24) and BUSCO completeness scores (98.40% for assembly, 98.60% for annotation; Fig. S3), representing a significant advance over previously reported *Rhododendron* genomes (Zhang et al., 2017; Wen et al., 2025a; Wang et al., 2025).

**Fig. 1:**
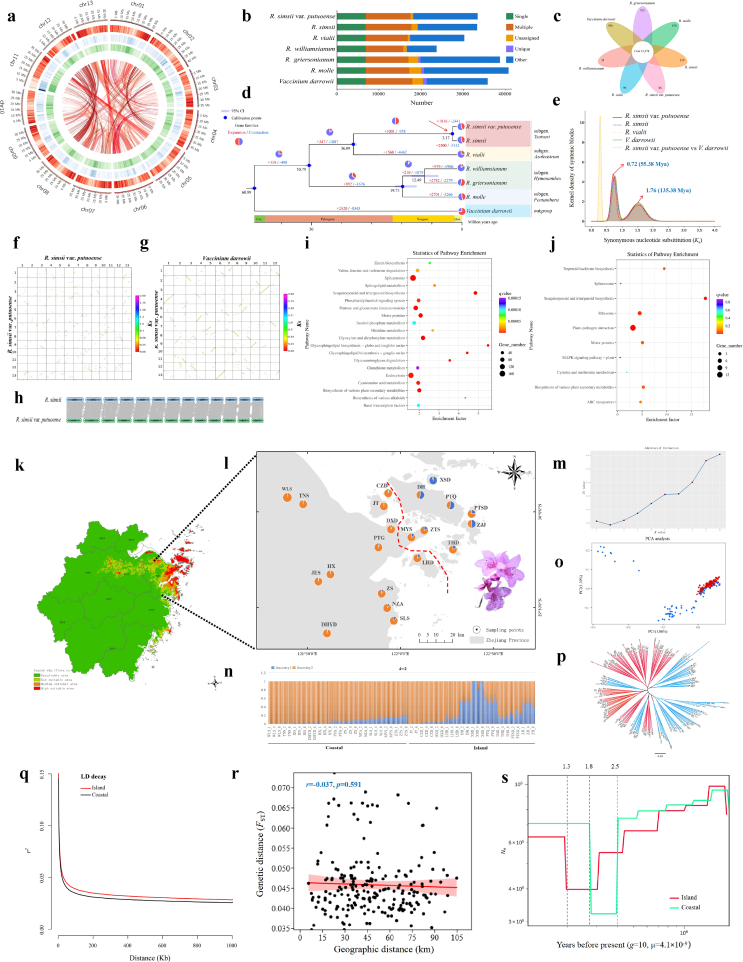
Genome assembly and population genomics of *Rhododendron simsii* var*. putuoense.***a:** Genome features of the chromosome-scale genome. **b:** Distribution of orthologous genes among six closely related species within the Ericaceae family. **c:** Clustering petal map showing the numbers of core and unique gene families. **d:** Phylogenetic tree and analysis of gene family expansions and contractions among six Ericaceae species: The scale at the bottom represents divergence time, with specific time estimates labeled adjacent to nodes. Lavender-colored bars denote 95% confidence intervals for divergence times. Blue nodes indicate fossil-calibrated time points, while red and blue numbers signify significantly expanded and contracted gene families, respectively. **e:** The *K*_s_ density plot of collinear genes within *R. simsii* var. *putuoense* and other related species. **f:** Collinearity within the genome of *R. simsii* var. *putuoense.***g:** Collinearity analysis between *R. simsii* var. *putuoense* and *Vacciniumdarrowii.***h:** Syntenic comparisons between *R. simsii* var. *putuoense* and *R. simsii.***i:** KEGG enrichment analysis for rapidly expanded gene families. **j:** KEGG enrichment analysis for rapidly contracted gene families. **k:** Potential distribution under the current climate scenario predicted by the MaxEnt model, with habitat suitability classified into four categories based on the habitat suitability index. **l:** Sampling locations of 21 natural populations, with pie charts illustrating the population structure according to ADMIXTURE analysis at the most likely cluster number *K* = 2. **m:** Distribution of CV error values corresponding to different *K* values. **n:** Histograms from the ADMIXTURE analysis showing individual ancestry assignments for 210 samples at *K* = 2, which represents the optimal value determined by cross-validation analysis. **o:** Principal component analysis (PCA). **p:** Unrooted phylogenetic tree. **q:** Pattern of linkage disequilibrium decay for the island and the coastal groups. **r:** Geographical effects on genetic variation revealed by Mantel tests. **s:** Demographic history of *R. simsii* var. *putuoense* reconstructed via the Pairwise Sequentially Markovian Coalescent (PSMC) model: PSMC plot showing changes in effective population size (*N*_e_) over time.

Repetitive sequences accounted for 49.35% of the genome (236,527,650 bp), dominated by unknown repeats (22.21%, 106,469,217 bp) and LTRs (18.06%, 86,583,678 bp). They are followed by DNA transposons (6.14%), satellites (0.12%), and SINEs (0.09%; Table S3). These sequences drive genetic variation and serve as effective molecular markers, as IRAP markers derived from retrotransposons have been shown to efficiently assess genetic diversity and stability in *Rhododendron* (Wen et al., 2023). To validate the gene annotation, we first analyzed the gene structure and number across *R. simsii* var. *putuoense* and six closely related species: *R. griersonianum* (Ma et al., 2021), *R. molle* (Nie et al., 2023), *R. simsii* (Yang et al., 2020), *R. vialii* (Chang et al., 2023), *R. williamsianum* (Soza et al., 2019), and *Vaccinium* *darrowii* (Yu et al., 2021). The distributions of exons and introns were generally consistent among species, all exhibiting skewed patterns, although variations in gene length and CDS length were observed, particularly in the genomes of *R. griersonianum* and *R. molle* (Fig. S4). Gene annotation integrating *de novo*, homology-based, and RNA-Seq-assisted predictions identified 33,248 protein-coding genes with average lengths of CDS (1261 bp), exons (326 bp) and introns (876 bp) (Table S4). Functional annotation against six databases (COG, GO, KEGG, KOG, Swiss-Prot, and NR) assigned functions to 31,074 genes, including 12,594 (300–1000 bp) and 16,994 (> 1000 bp) genes (Table S5).

Comparative genomic analysis of *Rhododendron simsii* var. *putuoense* and six closely related plants revealed 12,278 shared gene families, with 88 unique to *R. simsii* var. *putuoense* (Fig. 1c). Consistent with ddRAD-seq (Zhu et al., 2025) and cpDNA (Zhu and Lv, 2025), the phylogenetic tree, reconstructed from seven species using 6889 single-copy and 10,613 multiple-copy orthologs (Fig. 1b and Table S6), confirms that *R. simsii* var. *putuoense* is the closest relative of *R. simsii,* estimating their divergence time around 3.17 million years ago (Mya) (Fig. 1d). Gene family analysis identified 1816 expanded and 2441 contracted families (Fig. 1e). Functional enrichment analysis revealed distinct pathway-specific patterns, providing insights into adaptive strategies to coastal environments. Expanded families were significantly enriched in two key functional pathways: the sesquiterpenoid and triterpenoid biosynthesis pathway associated with plant secondary metabolite production, and spliceosomes (Fig. 1i). Enrichment for these genes indicates functional specialization: the former may enhance abiotic and biotic stress resistance (e.g., drought, pathogens) through increased synthesis of secondary metabolites, while the latter may improve RNA splicing efficiency, enabling rapid responses to environmental signals (Ma et al., 2024; Alhabsi et al., 2025). In contrast, contracted families were linked to the terpenoid backbone biosynthesis pathway and plant-pathogen interaction genes (Fig. 1j), suggesting a resource allocation trade-off: the reduction in general terpenoid precursors may redirect carbon flux toward specialized metabolites (e.g., flavonoids) (Saadat et al., 2023), which are critical for stress resistance in coastal habitats. Together, the coordinated expansion of pathways involved in specialized metabolism and the contraction of conserved metabolic and defense-related pathways underscores a genomic mechanism underlying niche adaptation in *Rhododendron* species. As seen in the karst-endemic *R. bailiense*, amplification of TPS and CYP gene families provides a genetic foundation for enhanced production of stress-mitigating compounds (e.g., flavonoids and terpenoids), while optimization of the spliceosome machinery supports dynamic gene regulation in fluctuating environments (Wen et al., 2025b). This evolutionary pattern reinforces the hypothesis that divergence in secondary metabolism plays a pivotal role in adaptive radiation within marginal habitats.

Whole-genome duplication (WGD) is known as a key driver of plant evolution and a recognized adaptive mechanism for angiosperms responding to environmental challenges (Soltis and Soltis, 2016). Based on the collinearity relationship between different regions within the *Rhododendron simsii* var. *putuoense* (Fig. 1f) and between closely related species (Fig. 1g and h), we calculated synonymous nucleotide substitution (*K*_s_) among collinear genes (Fig. 1e and Table S7). Two distinct peaks were detected with *K*_s_ values of approximately 0.72 and 1.76. These peaks are conserved across other species in the same genus, suggesting that *R. simsii* var. *putuoense* has retained traces of two shared ancient WGD events within the genus *Rhododendron*. Specifically, a relatively recent WGD event occurred approximately 55.38 Mya, which corresponds to the WGD-β event shared by some families within Ericales (Wang et al., 2021). An ancient event, dated to around 135.38 Mya, is consistent with an ancient whole-genome triplication (WGT-γ) event that is commonly observed among core eudicots (Badouin et al., 2017).

Admixture analysis (*K* = 2, lowest CV error; Fig. 1m) divided 21 populations into two genetic clusters (Fig. 1l and n), but PCA and the Splits Tree phylogenetic network showed extensive intermixing between island and coastal groups (e.g., coastal populations MYS/ZTS clustered with island groups; Fig. 1o–p). This incomplete lineage sorting aligns with the archipelago's young geological age (∼8500 years of isolation) or recent secondary contact, limiting lineage-specific mutations (Cros et al., 2020).

The species exhibits moderate genetic diversity (π = 0.2666, *H*_*o*_ = 0.2533, *H*_e_ = 0.2653), higher than *R. amesiae*-*R. concinnum* (π = 0.123, *H*_*o*_ = 0.041, *H*_e_ = 0.111; Ao et al., 2022), slightly higher than *R. bailiense* (π = 0.2489, *H*_o_ = 0.2039, *H*_e_ = 0.2331; Luo et al., 2025), but lower than *R. henanense* subsp. *lingbaoense* (π = 0.3768, *H*_*o*_ = 0.6035, *H*_e_ = 0.3521; Zhou et al., 2023). Island and coastal groups showed similar diversity indices (*H*_o_, *H*_e_, π), but island populations had higher *F*_IS_ (0.0735), indicating increased inbreeding risk (Table S8).

Pairwise Sequentially Markovian Coalescent (PSMC) analysis revealed stable effective population sizes (*N*_e_) initially, followed by a synchronous bottleneck ∼2.5 Mya, with asynchronous recovery: coastal populations rebounded ∼1.8 Mya, and island populations ∼1.3 Mya (Fig. 1u), coinciding with regional sea-level regression during the mid-Pleistocene, which connected the Zhoushan Archipelago to the mainland (Zhang et al., 2025). The linkage disequilibrium (LD) decayed more slowly in island groups (*r*^2^ stabilized at 0.04), reflecting stronger isolation, while Mantel tests showed no significant correlation between genetic differentiation (*F*_ST_) and geographical distance (*r* = −0.037, *P* = 0.591; Fig. 1q and r).

MaxEnt models (AUC = 0.9998/0.9994 for training/test data) identified precipitation seasonality (bio15, 48.28%) and coldest month minimum temperature (bio6, 21.53%) as key distribution limiting factor, with high-suitability areas in the Zhoushan Archipelago and adjacent coasts (Figs. 1k and S3). Selective sweep analysis identified 278 regions (6889 genes), enriched in molecular function (MF) categories and pathways related to metabolism, cell signal transduction, and programmed cell death (Fig. S6a–c), indicating adaptive selection for coastal stress tolerance (e.g., salt, cold, drought).

In conclusion, we present the first chromosome-level genome assembly of *R. simsii* var. *putuoense* (479.30 Mb, contig N50 = 33.97 Mb), with 99.91% sequences anchored to 13 pseudo-chromosomes, and retaining two ancient whole-genome duplication events (WGD-β: ∼55.38 Mya; WGT-γ: ∼135.38 Mya). Population genomic analyses of 21 populations (10 individuals each, 210 total) reveal a coastal-island genetic structure with incomplete lineage sorting and ongoing gene flow, reflecting the archipelago's young geological history. Demographic inference shows a synchronous bottleneck (∼2.5 Mya) followed by asynchronous recovery (coastal: ∼1.8 Mya; island: ∼1.3 Mya), leading to weak genetic differentiation. Island populations exhibit stronger selection pressure, with expanded secondary metabolite biosynthesis gene families and positive selection on salt/cold tolerance genes, elucidating coastal adaptation. This study fills coastal *Rhododendron* genomics gaps, reveals a “divergence-with-gene-flow” pattern in land-bridge island plants, and informs conservation and horticultural utilization of this endemic species.

## CRediT authorship contribution statement

Hong Zhu: Writing – original draft, Writing – review & editing, Visualization, Methodology, Formal analysis, Data curation, Conceptualization, Funding acquisition. Haojie Gao: Resources, Investigation, Formal analysis. Hepeng Li: Writing – review & editing, Supervision, Resources, Project administration.

## Data availability

The genome assembly and raw sequence data have been submitted to the national Center for Biotechnology Information (NCBI) database under the accession JBQRKF000000000 (BioProject ID: PRJNA1305678).

## Declaration of competing interest

The authors declare that they have no known competing financial interests or personal relationships that could have appeared to influence the work reported in this paper.
