# Supplementary material for: Chromosome-level genome and population genomics reveal demographic history, incomplete divergence and coastal adaptation of Rhododendron simsii var. putuoense in East China
Source: Plant Divers. 2025 Dec 10;48(3):635–9. doi: 10.1016/j.pld.2025.12.002 (PMC13250295; doi:10.1016/j.pld.2025.12.002)
Supplement: Multimedia component 1 [file mmc1.docx]

**Chromosome-level genome and population genomics reveal demographic history, incomplete divergence and coastal adaptation of *Rhododendron simsii* var. *putuoense* in East China**

Hong Zhu (朱弘)^a^，Haojie Gao (高浩杰)^b^，Hepeng Li (李贺鹏)^a,*^

^a^ *Zhejiang Academy of Forestry, Research Centre for Zhejiang Wetland, Hangzhou 310023, China*

^b^ *Zhoushan Academy of Forestry, Zhoushan 316000, China*

**First author:**

Hong Zhu (E-mail: 1107410987@qq.com; ORCID: 0000-0003-4048-2748)

***Corresponding author:**

Hepeng Li (E-mail: 84244@163.com; ORCID: 0009-0008-1593-7046)

**Supplementary Figures**


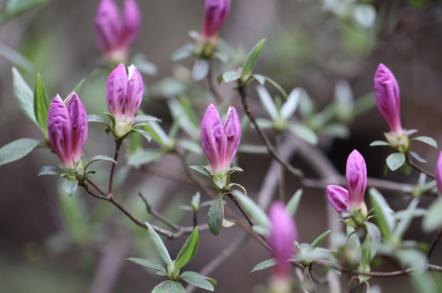

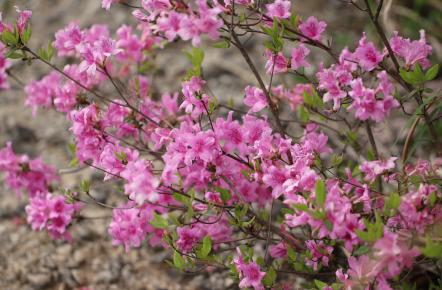

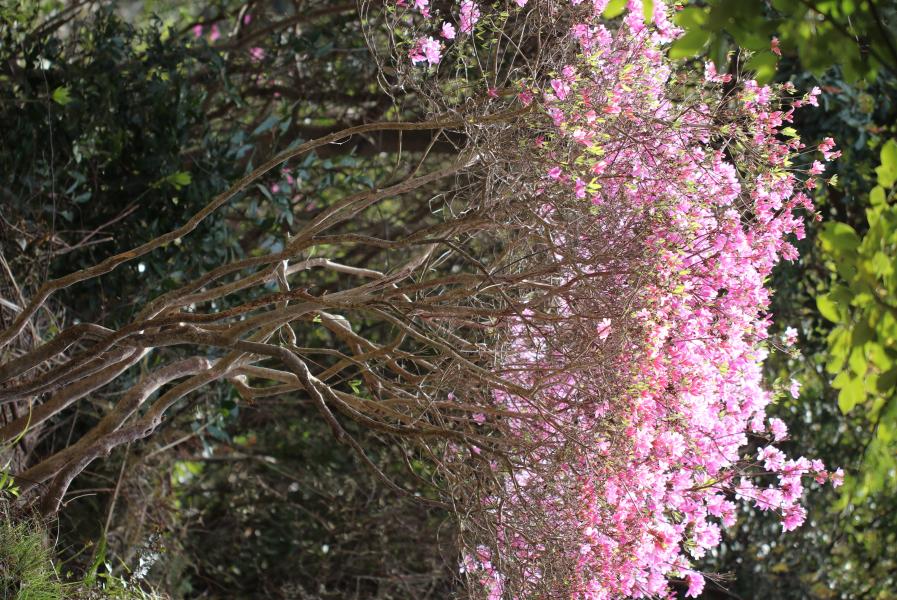

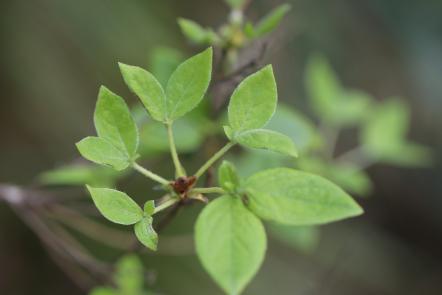


**a**

**b**

**c**

**d**

Fig. S1. Plant morphological characteristics of *Rhododendron simsii* var. *putuoense*. **a:** Whole individual with **b:** Terminal young leaves grow in early spring, **c:** Early spring flower buds and **d:** Inflorescence.


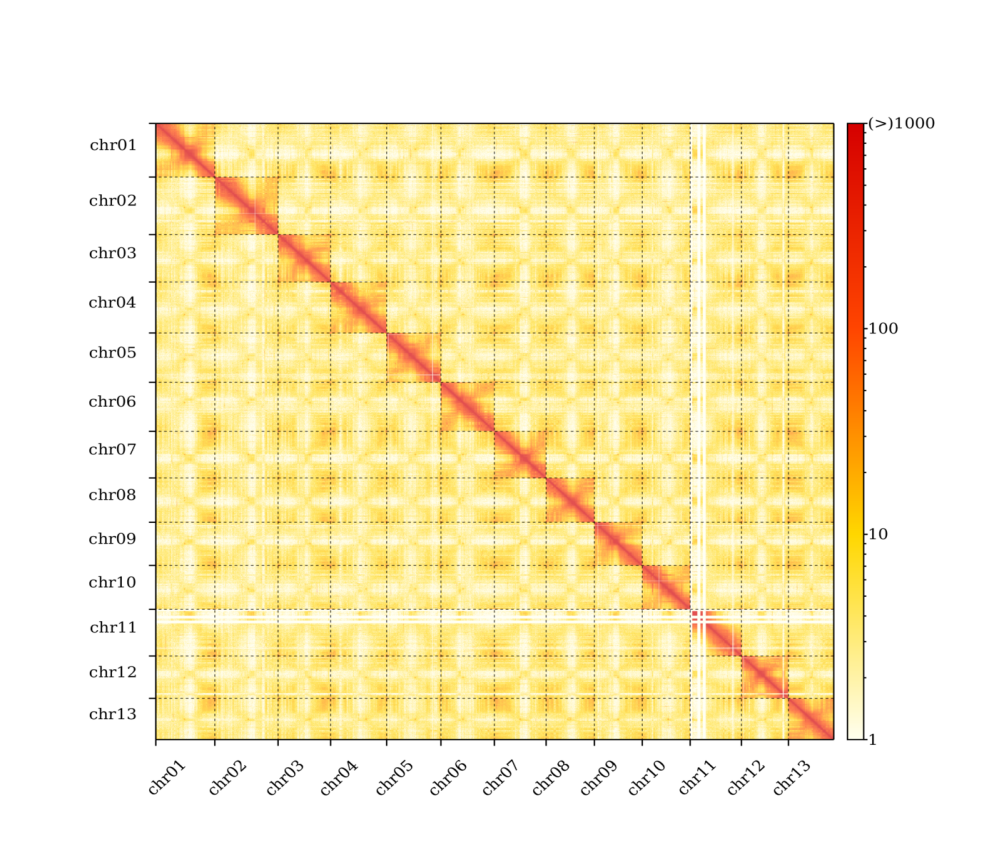


Fig. S2. Heatmap of 13 chromosome interactions with Hi-C data for *R. simsii* var. *putuoense.* .


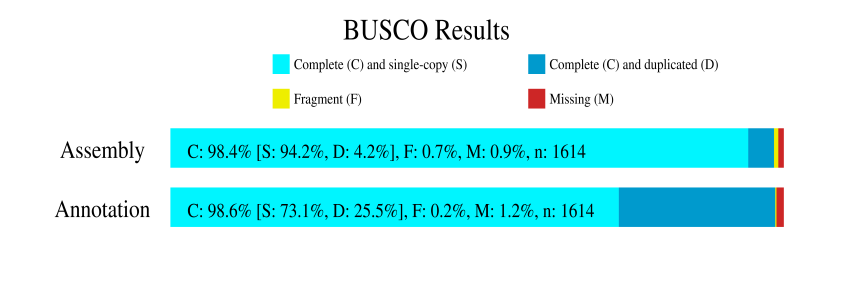


Fig. S3. Benchmarking Universal Single-Copy Orthologs (BUSCO) assessment results for *R. simsii* var. *putuoense* of genome assembly results.


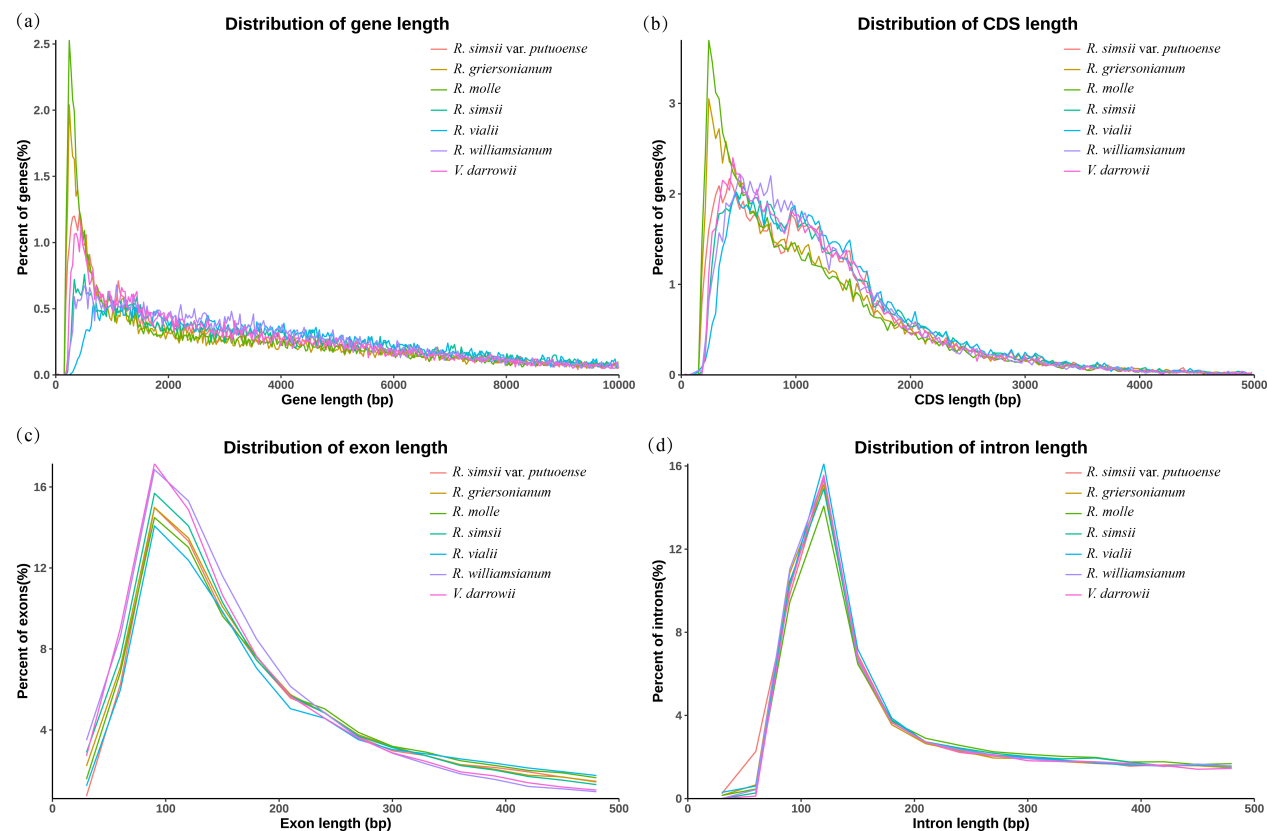
Fig. S4. Annotation quality comparison of *R. simsii* var. *putuoense* and six closely related species within the Ericaceae family. **a:** gene length distribution. **b:** CDS length distribution. **c:** exon length distribution and **d:** intron length distribution. The y-axis represents the percentage of genes of a certain statistical length in the total number of genes.

Fig. S5. Results of the contribution of training parameters and environmental factors, output from the Plant Distribution Prediction Platform for China (PPDC).


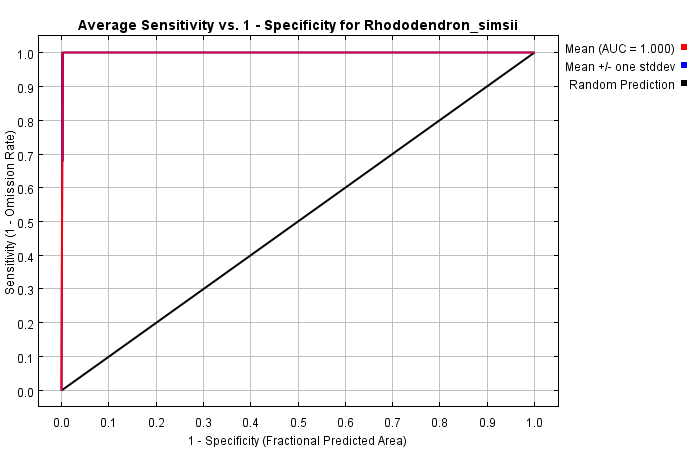

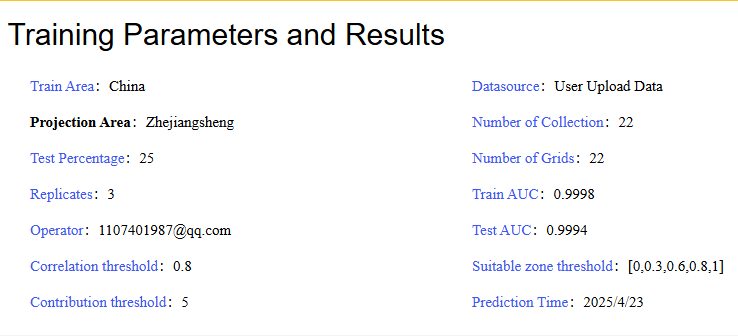

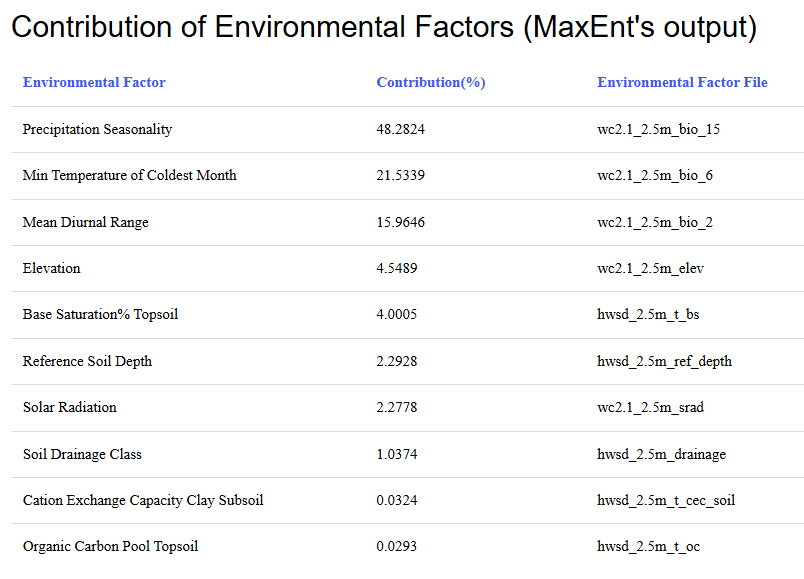


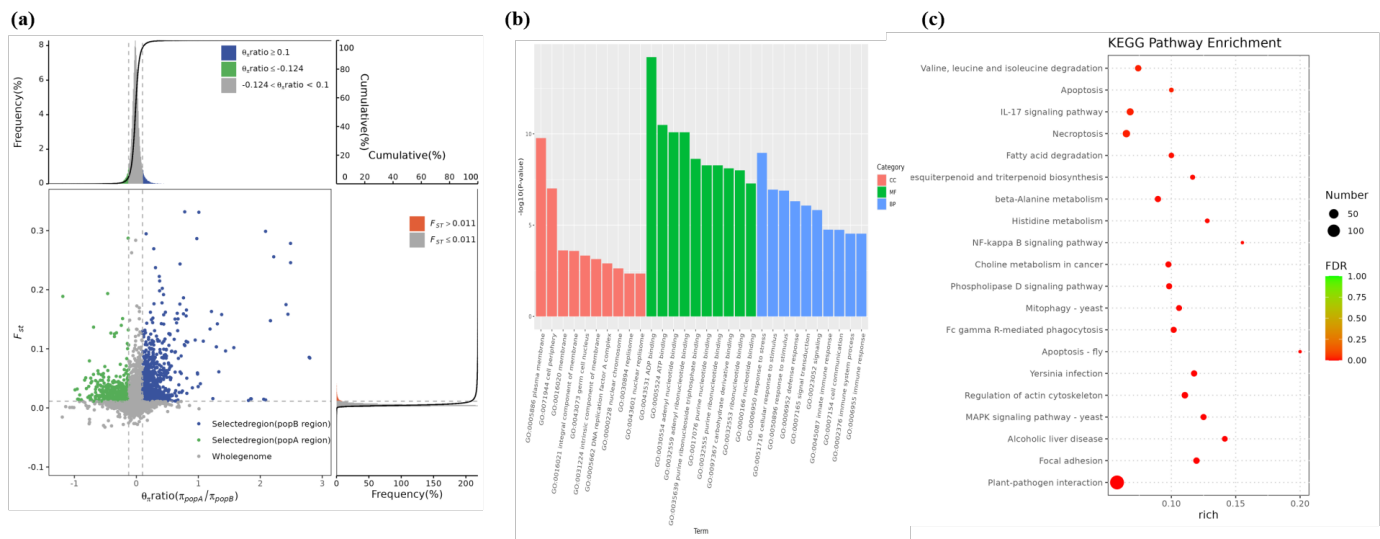
Fig. S6. Genome selection signal analysis. **a:** Gene function enrichment analysis of selection sweep regions. **b:** The GO enrichment analysis and **c:** The KEGG enrichment analysis.

**Supplementary Tables**

| Table S1. Statistics for the sequencing data of *Rhododendron simsii* var. *putuoense* genome. | | | | | | | | |
| --- | --- | --- | --- | --- | --- | --- | --- | --- |
| **Type** | **Platform** | **Numbers of Read** | **Numbers of Base** | **Mean Read Length** | **N50 Read Length** | **GC(%)** | **Q20(%)** | **Q30(%)** |
| Hi-C | Illumina NovaSeq 6000 | 247,700,195 | 74,224,718,018 | __ | __ | 40.86 | 98.65 | 95.78 |
| RNA | PromethION 48 | 39,277,262 | 11,783,178,600 | __ | __ | 46.37 | 98.49 | 94.05 |
| HiFi | PacBio Sequel II | 1,622,590 | 34,269,081,538 | 21,119 | 21,174 | __ | __ | __ |

| Table S2. Statistics of chromosome level assembly of *R. simsii* var. *putuoense*. | | |
| --- | --- | --- |
| **Chromosome** | **Length (bp)** | **Contig number** |
| chr01 | 41,725,171 | 1 |
| chr02 | 44,636,791 | 2 |
| chr03 | 36,952,499 | 1 |
| chr04 | 39,593,499 | 1 |
| chr05 | 38,391,532 | 2 |
| chr06 | 37,832,597 | 2 |
| chr07 | 36,500,050 | 1 |
| chr08 | 34,165,448 | 1 |
| chr09 | 33,752,523 | 1 |
| chr10 | 33,966,205 | 1 |
| chr11 | 36,226,484 | 3 |
| chr12 | 33,053,004 | 1 |
| chr13 | 32,056,792 | 1 |
| chrUn | 449,936 (0.09%) | 2 |
| Chr | 478,852,595 (99.91%) | 18 |
| Total | 479,302,531 | 20 |

| Table S3. Statistics of repeated sequences prediction results in the *R. simsii* var. *putuoense* genome. | | |
| --- | --- | --- |
| **Type** | **Length (bp)** | **Percentage of the genome (%)** |
| DNA transposon | 29,426,637 | 6.14 |
| LINE | 10,285,582 | 2.15 |
| SINE | 415,948 | 0.09 |
| LTR | 86,583,678 | 18.06 |
| Satellite | 583,786 | 0.12 |
| Other | 2,762,802 | 0.58 |
| Unknown | 106,469,217 | 22.21 |
| Total | 236,527,650 | 49.35 |

| Table S4. Statistics of protein-coding gene annotation. | | | | | | | | |
| --- | --- | --- | --- | --- | --- | --- | --- | --- |
| **Method** | **Software** | **Species** | **Gene number** | **Average gene length (bp)** | **Average CDS length (bp)** | **Average exon number per gene** | **Average exon length (bp)** | **Average intron length (bp)** |
| Ab initio | AUGUSTUS |  | 28,701 | 6,480 | 1,222 | 5.25 | 232 | 1,237 |
| Ab initio | GlimmerHMM |  | 46,933 | 8,824 | 669 | 3.41 | 196 | 3,380 |
| Homology-based | GeMoMa | 1. *griersonianum* | 40,475 | 4,107 | 1,109 | 4.47 | 248 | 862 |
| Homology-based | GeMoMa | *R. molle* | 52,479 | 3,222 | 970 | 3.83 | 253 | 795 |
| Homology-based | GeMoMa | *R. simsii* | 33,027 | 4,872 | 1,275 | 5.47 | 233 | 804 |
| Homology-based | GeMoMa | *R. vialii* | 37,758 | 4,237 | 1,328 | 4.88 | 272 | 749 |
| Homology-based | GeMoMa | *R. williamsianum* | 25,040 | 4,776 | 1,234 | 5.42 | 227 | 801 |
| Homology-based | GeMoMa | *Vaccinium darrowii* | 32,474 | 4,450 | 1,260 | 4.95 | 254 | 807 |
| RNAseq | TransDecoder | NGS | 21,034 | 7,869 | 1,573 | 7.52 | 358 | 1,028 |
| Integration | EVM |  | 33,522 | 4,332 | 1,221 | 4.64 | 263 | 855 |
| Final set | PASA |  | 33,248 | 4,985 | 1,261 | 5.12 | 326 | 876 |

| Table S5. Summary of genes annotated with the COG, GO, KEGG, KOG, Swiss-Prot and NR databases. | | | | |
| --- | --- | --- | --- | --- |
| **Type** | **Database** | **Number** | **300<=length<1000** | **length>=1000** |
| Annotation | COG | 10,358 | 3,013 | 7,227 |
|  | GO | 20,391 | 7,113 | 12,745 |
|  | KEGG | 18,678 | 6,244 | 11,981 |
|  | KOG | 16,095 | 5,574 | 10,089 |
|  | Swiss-Prot | 22,429 | 7,803 | 14,028 |
|  | NR | 31,056 | 12,585 | 16,992 |
| Total | All annotated | 31,074 | 12,594 | 16,994 |

| Table S6. Statistics of gene family identification. | | | | | | | | |
| --- | --- | --- | --- | --- | --- | --- | --- | --- |
| **Samples** | **Total genes** | **Genes in families** | **Unassigned genes** | **Families** | **Unique families** | **Gene of single-copy orthologs** | **Gene of multiple-copy orthologs** | **Gene of other orthologs** |
| *Rhododendron griersonianum* | 38,146 | 35,778 | 2,368 | 21,745 | 513 | 6,889 | 10,124 | 18,765 |
| *R. molle* | 40,021 | 37,201 | 2,820 | 22,304 | 670 | 6,889 | 10,308 | 20,004 |
| *R. simsii* | 32,999 | 31,636 | 1,363 | 20,415 | 219 | 6,889 | 10,856 | 13,891 |
| *R. simsii* var. *putuoense* | 33,248 | 32,653 | 595 | 20,919 | 88 | 6,889 | 10,613 | 15,151 |
| *R. vialii* | 30,067 | 29,572 | 495 | 19,804 | 99 | 6,889 | 9,952 | 12,731 |
| *R. williamsianum* | 23,559 | 22,729 | 830 | 18,014 | 38 | 6,889 | 8,903 | 6,937 |
| *Vaccinium darrowii* | 34,806 | 32,340 | 2,466 | 19,044 | 936 | 6,889 | 11,065 | 14,386 |

| Table S7. The distribution of synonymous substitution rate (*K*_s_) for whole genome duplication (WGD) events. | | | | | | | | | |
| --- | --- | --- | --- | --- | --- | --- | --- | --- | --- |
| **Sample** | **WGD1-height** | **WGD1-mu** | **WGD1-wid** | **WGD2-height** | **WGD2-mu** | **WGD2-wid** | **WGD3-height** | **WGD3-mu** | **WGD3-wid** |
| Rsim.put | 4.730558308383791 | 0.7158699734426602 | 0.11174673955556803 | 1.7619109369936214 | 1.504744185917725 | 0.30970858274647267 |  |  |  |
| Rsim | 4.475422265161373 | 0.742322396403848 | 0.12704455089851768 | 1.6121923821920459 | 1.5268558960696434 | 0.3452805948773877 |  |  |  |
| Rvia | 4.813673884300428 | 0.7114300245070617 | 0.11226612880815838 | 1.7886217388923533 | 1.4869682651088285 | 0.31542176133395106 |  |  |  |
| Vdar | 4.613043140507545 | 0.7626103236102011 | 0.11899602848404903 | 1.8838830720844384 | 1.532518121542352 | 0.29274515052772054 |  |  |  |
| Rsim.put_Vdar | 10.675315633515332 | 0.2871224640344137 | 0.04651373319433894 | 4.266719055785605 | 0.764325040593101 | 0.1308860823701604 | 1.8348934987278298 | 1.5476344710624508 | 0.2983446141617735 |

| Table S8. Genetic diversity analysis between the island and the coastal group of *R. simsii* var. *putuoense.* | | | | | |
| --- | --- | --- | --- | --- | --- |
| **Group** | **Num Indv** | ***H_o_*** | ***H_e_*** | **π** | ***F*_IS_** |
| Island | 88.2406 | 0.2505 | 0.2656 | 0.2671 | 0.0735 |
| Coastal | 117.8840 | 0.2560 | 0.2649 | 0.2660 | 0.0525 |
| Average | 103.0623 | 0.2533 | 0.2653 | 0.2666 | 0.0630 |
| Num Indv, average number of nucleotide differences; *H*_o_, observed heterozygosity; *H*_e_, expected heterozygosity; π, nucleotide diversity; *F*_IS_, inbreeding coefﬁcient within population. | | | | | |

**Supplementary Materials and Methods**

**1. Sampling and sequencing**

Fresh leaves of *Rhododendron simsii* var. *putuoense* were collected from Wuleishan, Dinghai District, Zhoushan City, China (coordinates: 29.43°N, 122.20°E; altitude: 359 m) in March 2025. The sample was identified and collected by the authors, and a specimen was deposited in the Herbarium of Zhejiang Academy of Forestry (HZJAF, contact: Hong Zhu, Email: 1107401987@qq.com) under the voucher number HZ2025-DH-05.

A modified CTAB approach was used to extract high-molecular-weight DNA. The *Rhododendron simsii* var. *putuoense* genome was assembled by using integrated multiple sequencing and assembly technologies. The Illumina NovaSeq 6000 (Illumina, USA) and PacBio Sequel II platform (PacBio, USA) were applied for genomic sequencing to generate short and long genomic reads, respectively. From the high-throughput chromosome conformation capture (Hi-C) sequencing, 102.13 Gb clean data were obtained, containing 247.70 Mb of clean paired-end reads, and the GC content was 40.86% with Q20 and Q30 rates of 98.65% and 95.78%, respectively. The RNA-seq library (cDNA-PCR library) was sequenced using the PromethION 48 device (Oxford Nanopore Technologies, UK).

**2. De novo genome assembly, quality assessment and annotation**

For HiFi reads, assembly was performed using the hifiasm software (v0.25.0) (Cheng et al., 2021) with default parameters. The main contigs obtained were adjusted for subsequent analysis. The Juicer (v2.0) (Durand et al., 2016) and 3D-DNA software (Dudchenko et al., 2017) were used to cluster the contigs, and determine the tightness of associations between them. Then, JuicerTools (v3.0) (https://github.com/aidenlab/JuicerTools) was employed to convert the pairwise interaction relationships of contigs into a specified binary file (i.e., .hic file). Subsequently, the Juicebox (v2.15.07) (https://github.com/aidenlab/Juicebox) was used to manually correct the ordered and oriented contigs, thereby obtaining the final chromosome-level assembly result. The completeness of the assembled genome was assessed by calculating the benchmarking universal single-copy orthologs (BUSCO) score, employed in BUSCO (v5.2.1) (Simão et al., 2015).

*De novo* repetitive sequences were first generated from the genome assembly using RepeatModeler (v2.0.1) (http://www.repeatmasker.org/RepeatModeler/), including long interspersed nuclear elements (LINEs), short interspersed elements (SINEs), retrotransposons, long tandem repeats (LTRs), and helitrons found in DNA transposons. The predicted results were then integrated with the RepBase database (http://www.girinst.org/repbase) to enhance sequence annotation accuracy. Finally, RepeatMasker (v4.1.0) (http://www.repeatmasker.org) was employed to identify and characterize repetitive elements within the genome. Three independent methods, namely, *de novo* prediction, homology searching and RNA-Seq-based prediction, were used for gene prediction in the repeat-masked genome. Specifically, ab initio prediction was performed using AUGUSTUS (v3.3.3) and GlimmerHMM (v3.0.4) software (Majoros et al., 2004). For homology annotation, protein sequences of *R. griersonianum, R. molle, R. simsii, R. vialii, R. williamsianum* and *Vaccinium darrowii,* were aligned to the assembled genome using GeMoMa (v1.9) (Keilwagen et al., 2019). For RNA-Seq-based prediction, transcripts were reconstructed from RNA-seq data using StringTie (v2.1.3) (Pertea et al., 2015), and coding sequences were subsequently predicted with TransDecoder (v5.1.0) (https://github. com/TransDecoder/TransDecoder). The resulting gene predictions were integrated using EVidenceModeler (v1.1.1) (Haas et al., 2008), and the final dataset were updated with PASA (v2.5.2) (Haas et al., 2003). Functional annotations of protein-coding genes were compared with homologs in the COG (Clusters of Orthologous Groups), GO (gene ontology), KEGG (Kyoto Encyclopedia of Genes and Genomes), KOG (Eukaryotic Orthologous Groups), Swiss-Prot (Swiss Protein Institute), and NR (nonredundant proteins) databases using BLASTP (v2.10.1+). Domains and gene ontology terms were annotated through sequence comparisons via HMMER (v3.2.1) using Pfam (protein family) databases.

**3. Gene families and phylogenetic analyses**

The protein sequences of *Rhododendron simsii* var. *putuoense* and six other sequenced within the Ericaceae family, *R*. *simsii* (GCA_014282245.1) (Yang et al., 2020), *R. vialii* (GCF_030253575.1 (Chang et al., 2023), *R. molle* (GCA_025413875.1) (Nei et al., 2023), *R. williamsianum* (GCA_009746105.1) (Soza et al., 2019), *R. griersonianum* (GCA_018127125.1) (Ma et al., 2021), and *Vaccinium darrowii* (GCA_020921065.1) (Yu et al., 2021), were retrieved for the comparative analysis. OrthoFinder (v2.3.5) (Emms and Kelly, 2019) and Diamond (v2.0.6.144) (Buchfink et al., 2015) were used to search for and identify orthologous and paralogous gene groupings among the seven species to determine different gene families.

The single-copy genes from the OrthoFinder results were aligned using MAFFT (v7.4270), and then concatenated end-to-end. RAxML (v8.2.12) (Stamatakis, 2014) was subsequently employed for phylogenetic tree analysis with the PROTGAMMAJTT model and 1000 bootstrap replicate to generate a maximum likelihood species tree. A phylogenetic tree with divergence times was constructed using MCMCTree (v4.9h), part of the *PAML* package (Yang et al., 2007). The fossil calibration points were the *R. simsii-V. darrowii* divergence (68.99 million years ago, Mya), *R. simsii-R. molle* divergence (50.79 Mya), *R. simsii-R. vialii* divergence (36.09 Mya), and *R. molle-R. griersonianum* divergence (19.37 Mya) that were taken from the TimeTree database (Sanderford et al., 2022). To investigate potential expansion and contraction of gene families among different species, CAFE (v4.2.1) (De et al., 2006) was utilized, with gene families containing more than 100 genes excluded from the analysis.

For the shared single-copy genes identified from gene family analysis, positive selection signals were detected using PAML’s codeml tool (Model=2, NSsites=2) based on the Branch-site Model A. Posterior probabilities of each gene site were evaluated via Bayesian Empirical Bayes (BEB) analysis to screen for significant positively selected sites with Prob (ω>1) ≥ 0.95. The formula T = *K*_s_/2λ × 10^− 6^ Mya (Lynch and Conery, 2000) was used to estimate the timing of duplication events as divergence time (T) in millions of years ago (Mya), where λ =4.1× 10^− 9^ represents the average nucleotide substitution rate per year (Ma et al., 2021). To reveal the collinearity relationship between *R. simsii* var. *putuoense* and *R*. *simsii*, genome-wide synteny analysis was performed using the MCScanX toolkit (https://github.com/wyp1125/MCScanX) with default parameters.

**4. Population resequencing and SNP calling**

For whole-genome resequencing, a total of 210 wild accessions of *R. simsii* var. *putuoense* were collected from 21 natural populations (10 individuals each) across its natural distribution range in Eastern China. Genomic DNA was extracted from fresh young leaves using the CTAB method, and the quality of the extraction was evaluated by 0.8% agarose gel electrophoresis. Sequencing libraries with an average insert size of 400 bp were constructed, and paired-ends sequencing (150 bp read length) was performed on Illumina NovaSeq 6000 platform. Raw reads were filtered using Fastp (v0.20.0) with a sliding window method (Chen et al., 2018). High-quality filtered data were aligned to the *R. simsii* var. *putuoense* reference genome using the mem algorithm of BWA (v0.7.12-r1039) (Li and Durbin, 2009). To reduce PCR duplicates and optical duplicates, SAMtools (v1.16.1) (Li et al., 2009) software was used to sort the SAM files and convert them to BAM files, and GATK toolkit (Zhu et al., 2015) was utilized for SNP realignment. For the population SNP file, VCFtools (v0.1.17) (Danecek et al., 2011) was used to retain biallelic genotype sites, while filtering for sites with a minor allele frequency greater than 0.05 and a SNP missing rate less than 20% for subsequent analysis. ANNOVAR (Wang et al., 2010) was used for the annotation of the SNP.

**5. Population structure and genomic diversity**

The population genetic structure was analyzed using ADMIXTURE software (Alexander et al., 2009) with SNP data, setting *K*=1–10 (assuming 1–10 ancestral populations) under the admixture model, and other parameters followed default settings. The optimal *K* value closest to the true number of ancestral populations was determined based on cross-validation (CV) error values across different *K*. An unrooted phylogenetic tree was constructed using the Maximum Likelihood (ML) algorithm in SplitsTree4 (v4.19.2) software (Huson et al., 2006) to visualize evolutionary relationships and genetic relatedness among different populations. Principal Component Analysis (PCA) was performed using GCTA software (Yang et al., 2011) after excluding SNPs with minor allele frequency (MAF) less than 0.05, clustering individuals into distinct subgroups based on principal components.

**6. Demographic history, Mantel test, and LD decay**

Demographic history inference was conducted using the Pairwise Sequentially Markovian Coalescent (PSMC) model (Li and Durbin, 2011) to reconstruct fluctuations in effective population size (*N*_e_) over time. The mutation rate (μ) of *Rhododendron simsii* var. *putuoense* was set as 4.1×10^⁻9^ substitutions per site per generation and a generation time of 10 years according to Ma et al. (2021).

To evaluate the role of geographic factors in shaping genetic structure, isolation-by-distance (IBD) patterns were examined using Mantel test. Genetic distance was quantified by pairwise population *F*_ST_ values, while the geographic distance matrices were generated from sampling coordinates using an online tool (https://www.91gjx.com/index/index/distance). Linear regression and visualization of the relationship between genetic and geographic distances were performed in PAST5 software (v5.0.1) (Hammer, et al., 2001).

To compare genome-wide linkage disequilibrium (LD) dynamics between coastal and island group, LD decay patterns were patterns using PopLDdecay (v3.42) (Zhang et al., 2019). The coefficient of determination (*r*²) between pairwise loci was used to measure LD strength, and decay curves were generated to characterize differences in LD decay rates between the two groups.

**7. Species distribution predicting**

To rapidly assess the distribution of *Rhododendron simsii* var. *putuoense,* the Plant Distribution Prediction Platform for China (PPDC) (Qiu et al., 2024), a MaxEnt-based online tool, was employed for species distribution modeling. The platform integrates 19 general bioclimatic variables (Bio1–Bio19) with seven additional environmental factors. For model training, 22 occurrence records were compiled: 21 from field surveys and one from Xiazhi Island. To minimize multicollinearity, environmental variables with a correlation coefficient > 0.8 and a contribution rate < 5% were excluded. The jackknife method was used to evaluate variable contributions and identify key environmental factors influencing the species’ distribution. Based on model outputs, potential distribution area was classified into four suitability categories: unsuitable (0.0–0.3), low suitable (0.3–0.6), medium suitable (0.6–0.8), and high suitable (0.8–1.0).

**8. Selective sweep and gene function enrichment**

To detect genomic regions under strong selective sweeps, the distributions of θπ (also known as nucleotide diversity) and genetic differentiation among populations (*F*_ST_) values were analyzed by the Pixy (v1.0.0) (Korunes and Samuk, 2021). Regions with extremely low or high θπ ratios (5% left and right tails) and significantly high *F*_ST_ values (i.e., top 5% of *F*_ST_values) were selected as regions under strong selective sweeps.

References

Alexander, D.H., Novembre, J., Lange, K., 2009. Fast model-based estimation of ancestry in unrelated individuals. Genome Res. 19, 1655-1664.

Alhabsi, A., Ling, Y., Crespi, M., et al., 2025. Alternative splicing dynamics in plant adaptive responses to stress. Annu. Rev. Plant Biol. 76, 687-717.

Badouin, H., Gouzy, J., Grassa, C.J., et al., 2017. The sunflower genome provides insights into oil metabolism, flowering and Asterid evolution. Nature 546, 148-152.

Buchfink, B., Xie, C., Huson, D.H., 2015. Fast and sensitive protein alignment using DIAMOND. Nat. Methods, 12, 59-60.

Chang, Y., Zhang, R., Ma, Y., et al., 2023. A haplotype-resolved genome assembly of *Rhododendron vialii* based on PacBio HiFi reads and Hi-C data. Sci. Data, 10, 451.

Chen, S., Zhou, Y., Chen, Y., et al., 2018. fastp: an ultra-fast all-in-one FASTQ preprocessor. Bioinformatics 34, i884-i890.

Cheng, H., Concepcion, G.T., Feng, X., et al., 2021. Haplotype-resolved *de novo* assembly using phased assembly graphs with hifiasm. Nat. Methods, 18, 170-175.

Cros, E., Chattopadhyay, B., Garg, K. M., et al., 2020. Quaternary land bridges have not been universal conduits of gene flow. Mol. Ecol. 29, 2692-2706.

Danecek, P., Auton, A., Abecasis, G., et al., 2011. The variant call format and VCFtools. Bioinformatics 27, 2156-2158.

De Bie, T., Cristianini, N., Demuth, J.P., et al., 2006. CAFE: a computational tool for the study of gene family evolution. Bioinformatics 22, 1269-1271.

Dudchenko, O., Batra, S.S., Omer, A.D., et al., 2017. *De novo* assembly of the *Aedes aegypti* genome using Hi-C yields chromosome-length scaffolds. Science 356, 92-95.

Durand, N.C., Shamim, M.S., Machol, I., et al., 2016. Juicer provides a one-click system for analyzing loop-resolution Hi-C experiments. Cell Syst., 3, 95-98.

Emms, D. M., Kelly, S., 2019. OrthoFinder: phylogenetic orthology inference for comparative genomics. Genome Biol., 20, 238.

Hammer, Ø., Harper, D.A.T., Ryan, P.D., 2001. Past: paleontological statistics software package for educaton and data anlysis. Palaeontol. Electron. 4, 9.

Haas, B.J., Delcher, A.L., Mount, S.M., 2003. Improving the Arabidopsis genome annotation using maximal transcript alignment assemblies. Nucleic Acids Res. 31, 5654-5666.

Haas, B.J., Salzberg, S.L., Zhu, W., et al., 2008. Automated eukaryotic gene structure annotation using EVidenceModeler and the Program to Assemble Spliced Alignments. Genome Biol., 9, R7.

Huson, D.H., Bryant, D., 2006. Application of phylogenetic networks in evolutionary studies. Mol. Biol. Evol. 23, 254-267.

Jiang, K., Tong, X., Ding, Y.Q., et al., 2021. Shifting roles of the East China Sea in the phylogeography of red nanmu in East Asia. J. Biogeogr. 48, 2486-2501.

Jin, D.P., Lee, J.H., Xu, B., et al., 2016. Phylogeography of East Asian *Lespedeza buergeri* (Fabaceae) based on chloroplast and nuclear ribosomal DNA sequence variations. J. Plant Res. 129, 793-805.

Keilwagen, J., Hartung, F., Grau, J., 2019. GeMoMa: homology-based gene prediction utilizing intron position conservation and RNA-seq data. Methods Mol. Biol. 1962:161-177.

Korunes, K.L., Samuk, K. 2021. pixy: unbiased estimation of nucleotide diversity and divergence in the presence of missing data. Mol. Ecol. Resour. 21, 1359-1368.

Li, H., Durbin, R., 2009. Fast and accurate short read alignment with Burrows–Wheeler transform. Bioinformatics 25, 1754-1760.

Li, H., Durbin, R., 2011. Inference of human population history from individual whole-genome sequences. Nature 475, 493–496.

Li, H., Handsaker, B., Wysoker, A., et al., 2009. The sequence alignment/map format and SAMtools. Bioinformatics 25, 2078-2079.

Li, G.Y., Chen, Z.H., Hu, J.F., et al., 2010. Two new plant varieties from Putuo Island, Zhejiang Province. J. Zhejiang A&F Univ. 27, 908–909.

Loretán, G., Rueda, E.C., Cabrera, J.M., et al., 2020. Geographical isolation and restricted gene flow drive speciation of *Aegla singularis* (Decapoda: Anomura: Aeglidae) in southern South America. Biol. J. Linn. Soc. 129, 177-189.

Luo, J., Yuan, C., Wang, H., et al., 2025. Study on the genetic diversity characteristics of the endemic plant *Rhododendron bailiense* in Guizhou, China based on SNP molecular markers. Ecol. Evol., 15, e70966.

Lynch, M., Conery, J.S. 2000. The evolutionary fate and consequences of duplicate genes. Science, 290, 1151-1151.

Ma, H., Liu, Y., Liu, D., et al., 2021. Chromosome‐level genome assembly and population genetic analysis of a critically endangered *Rhododendron* provide insights into its conservation. Plant J. 107, 1533-1545.

Ma, S., Sun, C., Su, W., et al., 2024. Transcriptomic and physiological analysis of *Atractylodes chinensis* in response to drought stress reveals the putative genes related to sesquiterpenoid biosynthesis. BMC Plant Biol. 24, 91.

Majoros, W.H., Pertea, M., Salzberg, S.L. 2004. TigrScan and GlimmerHMM: two open source ab initio eukaryotic gene-finders. Bioinformatics 20, 2878-2879.

Nie, S., Zhao, S.W., Shi, et al., 2023. Gapless genome assembly of azalea and multi-omics investigation into divergence between two species with distinct flower color. Hortic. Res. 10, uhac241.

Pertea, M., Pertea, G.M., Antonescu, C.M., et al., 2015. StringTie enables improved reconstruction of a transcriptome from RNA-seq reads. Nat. Biotechnol. 33, 290-295.

Qiu, J., Zhang, J., Wang, Y., et al., 2024. PPDC: an online platform for the prediction of plant distributions in China. J. Plant Ecol. 17, rtae094.

Sanderford, M., Li, M., Stecher, G., et al., 2022. TimeTree 5: an expanded resource for species divergence times. Mol. Biol. Evol. 39, msac174.

Soltis, P.S., Soltis, D.E., 2016. Ancient WGD events as drivers of key innovations in angiosperms. Curr. Opin. Plant Biol. 30, 159-165.

Soza, V.L., Lindsley, D., Waalkes, A., et al., 2019. The *Rhododendron* genome and chromosomal organization provide insight into shared whole-genome duplications across the heath family (Ericaceae). Genome Biol. Evol. 11, 3353-3371.

Simão, F.A., Waterhouse, R.M., Ioannidis, P., et al., 2015. BUSCO: assessing genome assembly and annotation completeness with single-copy orthologs. Bioinformatics 31, 3210-3212.

Stamatakis, A., 2014. RAxML version 8: a tool for phylogenetic analysis and post-analysis of large phylogenies. Bioinformatics 30, 1312-1313.

Upadhyay, R., Saini, R., Shukla, P. K., et al., 2025. Role of secondary metabolites in plant defense mechanisms: A molecular and biotechnological insights. Phytochem. Rev. 24, 953-983.

Wang, K., Li, M., Hakonarson, H., 2010. ANNOVAR: functional annotation of genetic variants from high-throughput sequencing data. Nucleic Acids Res. 38, e164-e164.

Wang, Z., Qin, K., Chen, W., et al., 2025. High-quality genome assembly and transcriptome of *Rhododendron platypodum* provide insights into its evolution and heat stress response. Plants 14, 1233.

Wang, X., Gao, Y., Wu, X., et al., 2021. High‐quality evergreen azalea genome reveals tandem duplication‐facilitated low‐altitude adaptability and floral scent evolution. Plant Biotechnol. J. 19, 2544-2560.

Wen, S., Zhao, H., Zhang, M., et al., 2023a. IRAPs in combination with highly informative ISSRs confer effective potentials for genetic diversity and fidelity assessment in *Rhododendron*. Int. J. Mol. Sci. 24, 6902.

Wen, S., Cai, X., Zhou, K., et al. 2025b. Metabolome and comparative genome provide insights into secondary metabolites generation of a rare karst‐growing *Rhododendron* in vitro culture. Plant J. 121, e17235.

Wen, S., Cai, X., Yang, K., et al., 2025. Chromosome‐level genome assembly of a rare karst‐growing *Rhododendron* species provides insights into its evolution and environmental adaptation. J. Syst. Evol. 63, 245-267.

Worsham, M.L., Julius, E.P., Nice, C.C., et al., 2017. Geographic isolation facilitates the evolution of reproductive isolation and morphological divergence. Ecol. Evol. 7, 10278-10288.

Xia, X.M., Du, H.L., Hu, X.D., et al., 2024. Genomic insights into adaptive evolution of the species-rich cosmopolitan plant genus *Rhododendron*. Cell Rep. 43,114745.

Yang, F. S., Nie, S., Liu, H., et al., 2020. Chromosome-level genome assembly of a parent species of widely cultivated azaleas. Nat. Commun., 11, 5269.

Yang, J., Lee, S.H., Goddard, M.E., et al., 2011. GCTA: a tool for genome-wide complex trait analysis. Am. J. Hum. Genet. 88, 76-82.

Yang, Z., 2007. PAML 4: phylogenetic analysis by maximum likelihood. Mol. Biol. Evol. 24, 1586-1591.

Yu, J., Hulse-Kemp, A.M., Babiker, E., et al., 2021. High-quality reference genome and annotation aids understanding of berry development for evergreen blueberry (*Vaccinium darrowii*). Hortic. Res. 8, 228.

Zhang, C., Dong, S.S., Xu, J.Y., et al., 2019. PopLDdecay: a fast and effective tool for linkage disequilibrium decay analysis based on variant call format files. Bioinformatics 35, 1786-1788.

Zhang, L., Xu, P., Cai, Y., et al., 2017. The draft genome assembly of *Rhododendron delavayi* Franch. var. *delavayi*. GigaScience 6, 1‑11.

Zhang, Z., Chen, W., Li, Z., et al., 2025. The island rule-like patterns of plant size variation in a young land-bridge archipelago: roles of environmental circumstance and biotic competition. Plant Divers. 47, 300-310.

Zhou, X., Zhang, C., Han, J. 2023. Genetic diversity of *Rhododendron henanense* subsp. *lingbaoense* revealed by whole-genome resequencing. Crop Breed. Appl. Biotechnol. 23, e439023112.

Zhu, P., He, L., Li, Y., et al., 2015. OTG-snpcaller: an optimized pipeline based on TMAP and GATK for SNP calling from ion torrent data. PLOS ONE 9, e97507.

Zhu, H., Li, D., Yue, C., et al., 2025. Development of single nucleotide polymorphism and phylogenetic analysis of *Rhododendron* species in Zhejiang Province, China, using ddRAD-Seq technology. Plants 14, 1548.
